# Novel avian paramyxovirus-based vaccine vectors expressing the Ebola virus glycoprotein elicit mucosal and humoral immune responses in guinea pigs

**DOI:** 10.1038/s41598-019-42004-4

**Published:** 2019-04-02

**Authors:** Asuka Yoshida, Shin-Hee Kim, Vinoth K. Manoharan, Berin P. Varghese, Anandan Paldurai, Siba K. Samal

**Affiliations:** Virginia-Maryland Regional College of Veterinary Medicine, University of Maryland, College Park, Maryland United States

## Abstract

Paramyxovirus vaccine vectors based on human parainfluenza virus type 3 (HPIV-3) and Newcastle disease virus (NDV) have been previously evaluated against Ebola virus (EBOV) challenge. Although both the viral vectored vaccines efficiently induce protective immunity, some concerns remain to be solved. Since HPIV-3 is a common human pathogen, the human population has pre-existing immunity to HPIV-3, which may restrict the replication of the vaccine vector. For NDV, mesogenic (intermediate virulent) strain used in previous studies is currently classified as a Select Agent in the United States, thus making it unsuitable to be used as a vaccine vector. To overcome these concerns, we have developed a modified NDV vector based on a mesogenic NDV strain, in which the ectodomains of envelope glycoproteins were replaced with the corresponding ectodomains from avian paramyxovirus serotype 3 (APMV-3). The modified NDV vector was highly attenuated in chickens and was able to express the EBOV glycoprotein (GP) gene at high level. In addition, the recombinant APMV-3 was also evaluated as a vaccine vector to express the EBOV GP gene. Guinea pigs immunized with these two vector vaccines developed high levels of neutralizing GP-specific IgG and IgA antibodies.

## Introduction

Ebola virus (EBOV) causes a severe, acute and often-fatal hemorrhagic fever in humans and non-human primates (NHPs)^1^. Currently, no licensed vaccines are available against EBOV, but several experimental vaccines are being investigated^2^. Recently, vectored vaccines were found to be protective in NHPs^2,3^. A number of experimental viral vector vaccines based on both RNA and DNA viruses have been evaluated^2,3^. However, each viral vector has both advantages and disadvantages. Among RNA viruses, paramyxoviruses have several features that make them suitable for use as vaccine vectors. Firstly, the complete cytoplasmic replication of paramyxoviruses avoids integration of their genomes into the host genome. Secondly, the genomes of paramyxoviruses do not undergo recombination, which makes these vectors genetically stable. Thirdly, paramyxoviruses can replicate in a variety of cell lines and infect various species of animals, which allows development of different types of viral vectors for different species of animals^4–7^.

The human parainfluenza virus type 3 (HPIV3) expressing EBOV glycoprotein (GP) was found to be immunogenic and protective against lethal EBOV challenge in NHPs^8^. Although, this vaccine was found to be effective after two doses of vaccination in HPIV3-immune NHPs, it was found that single dose of the vaccine induced substantially less immune response in HPIV3-immunised animals compared to HPIV3-naïve animals^9^. Therefore, it still remains unclear whether this vaccine will be effective in adult human population in presence of high levels of HPIV3 antibody. Furthermore, HPIV3 is a human pathogen and its safety in immunocompromised individuals is a concern. Therefore, there is a need to evaluate additional paramyxovirus vectors for development of an effective EBOV vaccine.

The avian paramyxoviruses (APMVs) are classified in the genus *Avulavirus* of the family *Paramyxoviridae* and are officially divided into thirteen serotypes (APMV-1 through APMV-13) based on hemagglutination inhibition (HI) and comparison of genetic distance^10,11^. Several additional APMV serotypes have been recently reported, which are awaiting official recognition^12,13^. APMV-1 includes all strains of Newcastle disease virus (NDV)^14^. NDV is an important pathogen of poultry. NDV strains are categorized into three pathotypes based on the severity of the disease in chickens: lentogenic (low virulent), mesogenic (moderately virulent), and velogenic (highly virulent)^14^. Lentogenic NDV strains have been widely used as live vaccines for the poultry. NDV is a good viral vector candidate for vaccine development because NDV infects almost all avian and non-avian species, including humans, and grows to high titer in embryonated eggs as well as in Vero cells, a WHO-approved cell line for vaccine production^7^. NDV-based EBOV vector vaccines have shown promising results in NHPs^15^. It has been reported that the mesogenic NDV strain Beaudette C (BC) replicates well in many tissue types of NHPs and elicits higher levels of immune responses compared to those induced by the lentogenic NDV strain LaSota^16^. However, all mesogenic and velogenic NDV strains are listed as Select Agents in the United States as they are pathogens of concern for the poultry, which makes them ineligible to be used as vaccine vectors. Therefore, an APMV vector used for human vaccine development should be safe for both poultry and humans in addition to being immunogenic for humans. Hence, the mesogenic NDV strain BC was modified to make it nonpathogenic for avian species. In addition, the potential of another APMV serotype for development of viral vector for humans has been evaluated.

Among the other APMV serotypes, APMV-3 is best suited as a vaccine vector for humans because it replicates better than the other APMV serotypes in a wide range of cell types, including Vero cells and human cell lines (Supplemental Fig. 1) and causes negligible disease associated with loss of egg production in turkeys and chickens^17,18^. APMV-3 is not a Select Agent. APMV-3 infects NHPs without causing any clinical disease signs^19^. This suggests that APMV-3 may be a safe vaccine vector in humans. A recombinant APMV-3 expressing green fluorescent protein (GFP) has been generated and the GFP gene was maintained for more than 10 passages^20^. Thus, APMV-3 represents a promising attenuated vector candidate for human vaccine development.

In order to keep the mesogenic NDV strain BC immunogenic in primates but nonpathogenic for avian species, the ectodomains of the F and HN protein genes of NDV strain BC were replaced with the corresponding ectodomains of APMV-3 strain Netherlands. These changes did not affect the virus replication remarkably but made the BC strain avirulent to chickens. We then inserted the EBOV GP gene into the modified NDV as well as to the recombinant APMV-3 (rAPMV-3). These modified NDV and rAPMV-3 vaccine candidates were evaluated *in vitro* in terms of their replication, GP expression and incorporation of GP into virus particles, while the induction of IgG and IgA responses and EBOV GP-specific neutralization antibodies response were evaluated in guinea pigs. Our results showed that not only the modified NDV vector but also the rAPMV-3 vector expressing GP are promising vaccine candidates for EBOV.

## Results

### Replacement of F and HN ectodomains of NDV by those of APMV-3 highly attenuated the virulence without affecting viral replication

**Figure 1:**
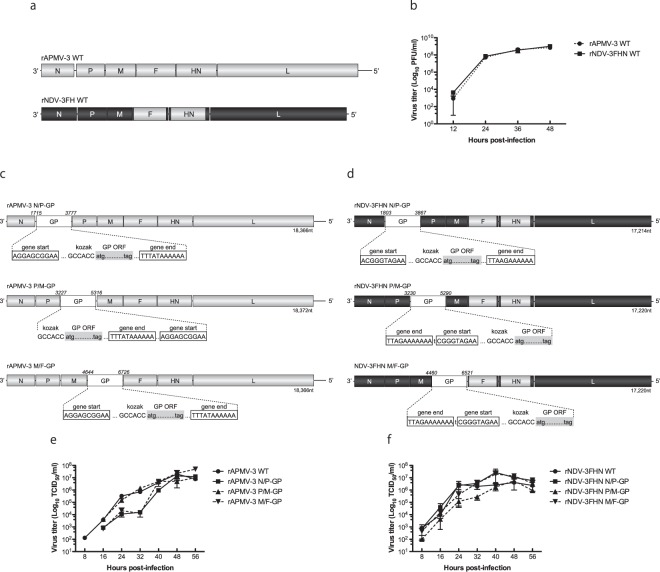
Multi-step growth kinetics of rNDV-3FHN and rAPMV-3 in embryonated chicken eggs and EBOV GP-expressing recombinants in DF-1 cells. (**a**) Schematic representation of rNDV-3FHN and rAPMV-3. The genes derived from NDV and APMV-3 are shown as black or gray respectively. (**b**) Multicycle growth kinetics of rNDV-3FHN and rAPMV-3. 10-day-old embryonated chicken eggs were inoculated with 100 PFU of each virus. Infectious allantoic fluids from eggs were harvested at 12 h intervals until 48 h p.i. and virus titers were determined at each time point. Results of two independent experiments are shown, and error bars show standard deviation. (**c**) and (**d**) Schematic representation of GP-expressing rNDV-3FHN and rAPMV-3. The EBOV GP gene was flanked by gene start and gene end signals of NDV or APMV-3 and inserted into indicated intergenic region of those antigenomic cDNA. (**e**) and (**f**) Multicycle growth kinetics of rNDV-3FHN and rAPMV-3. DF-1 cells in six-well plates were infected with parental and recombinant viruses at an M.O.I. of 0.01. Supernatants were collected at 8 h intervals until 56 h p.i. and virus titers were determined at each time point. Results of three independent experiments are shown, and error bars show standard deviation.

Use of mesogenic NDV strains as vaccine vectors is currently prohibited because of their potential to cause diseases in chickens. To circumvent this issue, we generated attenuated rNDV without affecting viral replication. The ectodomains of F and HN genes of mesogenic NDV strain BC were replaced with those of APMV-3 strain Netherlands (rNDV-3FHN) (Fig. 1a). The pathogenicity of the rNDV-3FHN to chickens was evaluated by mean death time (MDT) and by intracerebral pathogenicity index (ICPI) tests (Table 1). The MDT value of the rNDV-3FHN was more than 90 h, and the ICPI value was 0.08, indicating that this rNDV vector is avirulent in chickens.

**Table 1:** Pathogenicity of GP-expressing recombinant viruses in embryonated eggs and 1-day-old chicks.

Virus	MDT^a^ (h)
rNDV-3FH WT	>114
rAPMV-3 N/P-GP	>168
rNDV-3FH N/P-GP	>168
**Virus**	**ICPI** ^**b**^
rNDV-3FH WT	0.08
rAPMV-3 N/P-GP	0.63
rNDV-3FH N/P-GP	0.51

^a^Mean embryo death time (MDT): the mean time (h) for the minimum lethal dose of virus to kill all of the inoculated embryos^42^. Pathotype definition: virulent strains, <60 h; intermediate virulent strains, 60 to 90 h; avirulent strains, >90 h.

^b^Intracerebral pathogenicity index (ICPI): evaluation of disease and death following intracerebral inoculation in 1-day-old SPF chicks^42^. Pathotype definition: virulent strains, 1.5–2.0; intermediate virulent strains, 0.7–1.5; and avirulent strains, 0.0–0.7.

To examine whether the newly developed rNDV replicates efficiently compared to the parental APMV-3, we evaluated their growth kinetics in embryonated chicken eggs (Fig. 1b). The rNDV-3FHN replicated efficiently, reaching 1.02 × 10^9^ PFU/ml at 48 h p.i. Taken together, these results indicate that the newly developed rNDV can be used as an alternative vaccine vector.

### Position-dependent expression of GP by the rNDV-3FHN and rAPMV-3 vectors

In NDV, the optimum location for the foreign protein expression is known to be between the P and M genes of viral genome rather than the upstream position between the N and P genes^21–24^. Our recent study has also shown that the levels of expression of the foreign GFP was highest when inserted between the P and M genes^20^. In this study, we inserted EBOV GP gene into three different intergenic regions of both the rNDV-3FHN and the rAPMV-3 genomes (N-P, P-M, and M-F) (Fig. 1c,d). To examine whether replication of these recombinants was affected by the GP insertion, multi-step growth kinetics of these recovered viruses were compared with that of the non-GP-expressing parental viruses in DF-1 cells (Fig. 1e,f). At the final time point of 56 h p.i., there were no remarkable differences in viral titers between all of the GP-expressing recombinants and their respective parental viruses, all reaching titers of around 1 × 10^7^ TCID_50_/ml (Fig. 1e,f). For the GP-expressing rAPMV-3, their viral growth was slightly retarded at the early time point when the GP was inserted into the N-P and the M-F regions (Fig. 1e, P > 0.05), while among the GP-expressing rNDV-3FHN, no significant differences of growth kinetics were observed (Fig. 1f). These results indicated that the position of GP insertion did not largely affect virus growth.

**Figure 2:**
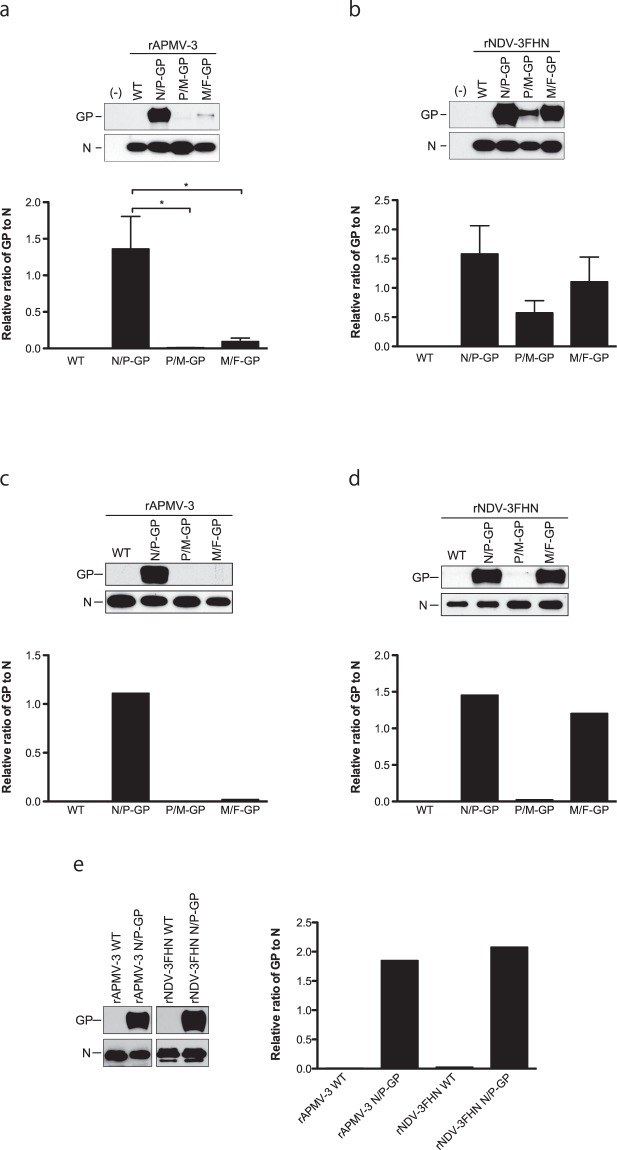
Expression and gene stability of EBOV GP by recombinants in DF-1 cells. (**a**) and (**b**) The level of GP expression at 24 h p.i. in DF-1 cells infected with indicated viruses was analyzed by Western blot. Amount of N proteins and GP proteins were quantitated and the ratio of GP to N is shown as bar graph. Results of three independent experiments are shown, and error bars show standard deviation. (**c**) and (**d**) The EBOV GP expressing rNDV-3FHN and rAPMV-3 were passaged ten times in DF-1 cells and (**e**) rNDV-3FHN N/P-GP and rAPMV-3 N/P-GP were passaged twenty times in DF-1 cells. The stability of GP expression was analyzed by Western blot. Amount of N proteins and GP proteins were quantitated and the ratio of GP to N is shown as bar graph. A result of single experiment is shown. *P < 0.05 by one-way ANOVA with Bonferroni post hoc test.

We then examined the expression levels of GP protein in DF-1 cells infected with the recombinant viruses (Fig. 2a,b). The size of protein bands detected was approximately 130 kDa corresponding to EBOV GP. The maximum expression of GP was observed when it was inserted between the N-P genes in both backbone viruses, following the 3′-to-5′ gradient attenuation of paramyxovirus transcription^25^. This is contradictory to our GFP recombinants of NDV-3FHN and APMV-3, which expressed maximum levels of GFP at the insertion location between the P-M genes (Supplemental Fig. 2 and^20^). Furthermore, in APMV-3 backbone viruses, the levels of GP expression were dramatically decreased at the insertion location between the P-M and between the M-F genes. In rNDV-3FHN vector, higher expression of the GP was found at insertion location between the M-F genes than between the P-M genes (P > 0.05). Taken together, our results show that in the case of EBOV GP, the gene expression did not follow the gradient transcription of *Paramyxoviridae* (Lamb and Parks, 2013), which is in contrast to a position-dependent expression of GFP by NDV and APMV-3^19,20,26^. Hence, the optimum location for a foreign protein expression might vary from backbone to backbone.

### The GP is incorporated into viral particles

**Figure 3:**
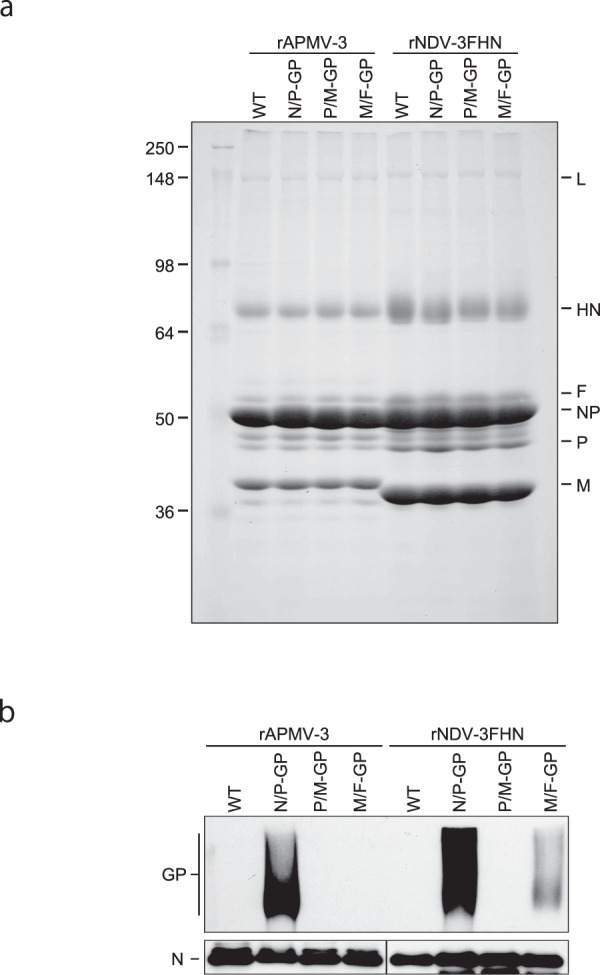
Incorporation of EBOV GP into virus particles. The viruses were harvested from allantoic fluids of infected eggs and purified through 30% sucrose cushion. (**a**) Purified virus particles were subjected to SDS-PAGE to analyze viral proteins profile by 1% CBB staining (**b**) Western blot was performed using anti-EBOV GP pAb to analyze incorporation of GP into virus particles. A result of single experiment is shown.

Previous studies have reported the incorporation of foreign viral transmembrane proteins into envelopes of rNDV particles^26–29^. To investigate whether GP was incorporated into the rNDV-3FHN and rAPMV-3 particles, we purified the recombinant viruses through a sucrose cushion (Fig. 3). The composition of virion proteins of both the rNDV-3FHN and rAPMV-3 recombinants was identical to that of their respective parental viruses (Fig. 3a), indicating that the insertion and expression of GP did not affect virion assembly. The GP was not clearly detected on the Coommassie Brilliant Blue (CBB)-stained gels, probably due to glycosylation with various degrees (Fig. 3a). However, the GP was clearly detected by Western blotting, as a broad band, due to the high level of glycosylation, as previously reported (Fig. 3b)^30^. These results confirm that the GP is efficiently incorporated into the viral particles of both the recombinants depending on the level of GP expression in the infected cells.

### GP expression of the recombinants were stable after consecutive passages

It has been reported that expression of the foreign genes from viral genomes is sometimes unstable, and suppressed during viral passages^31^. To evaluate stability of the GP expression of these recombinants, viruses were passaged through DF-1 cells up to ten times. After ten passages (P10), the expression levels of GP were similar between corresponding P3 and P10 recombinants (compare Fig. 2a–d). In case of rNDV-3FHN N/P-GP, M/F-GP, and rAPMV-3 N/P-GP, high levels of GP expression were still observed. However, in case of rNDV-3FHN P/M-GP, GP expression was diminished (Fig. 2c). The evaluation of GP expression of N/P-GP viruses after another ten passages (P20) did not show any decrease in the expression level of GP (Fig. 2e). The integrity of nucleotide sequences of the inserted GP gene as well as the entire genome of the N/P-GP recombinants were confirmed and no mutation was found. Furthermore, to determine the possibility of accumulation of mutant viruses which have unwanted mutations in the GP gene, GP gene of ten plaque-purified P20 virus clones were sequenced confirmed and no mutation was found.

### Pathogenicity of the GP-expressing recombinant viruses in chickens

Pathogenicity of rNDV-3FHN N/P-GP and rAPMV-3 N/P-GP recombinant viruses was evaluated by MDT and by ICPI tests (Table 1). The MDT values of the GP-expressing recombinants were greater than 90 h, and the ICPI value of rNDV N/P-GP and rAPMV-3 N/P-GP was 0.51 and 0.63 respectively, indicating that these recombinants are avirulent to chickens.

### The GP-expressing viruses induce the EBOV GP-specific antibodies in guinea pigs and those antibodies have neutralizing activity against GP expressing rVSVΔG-ZEBOV GP

**Figure 4:**
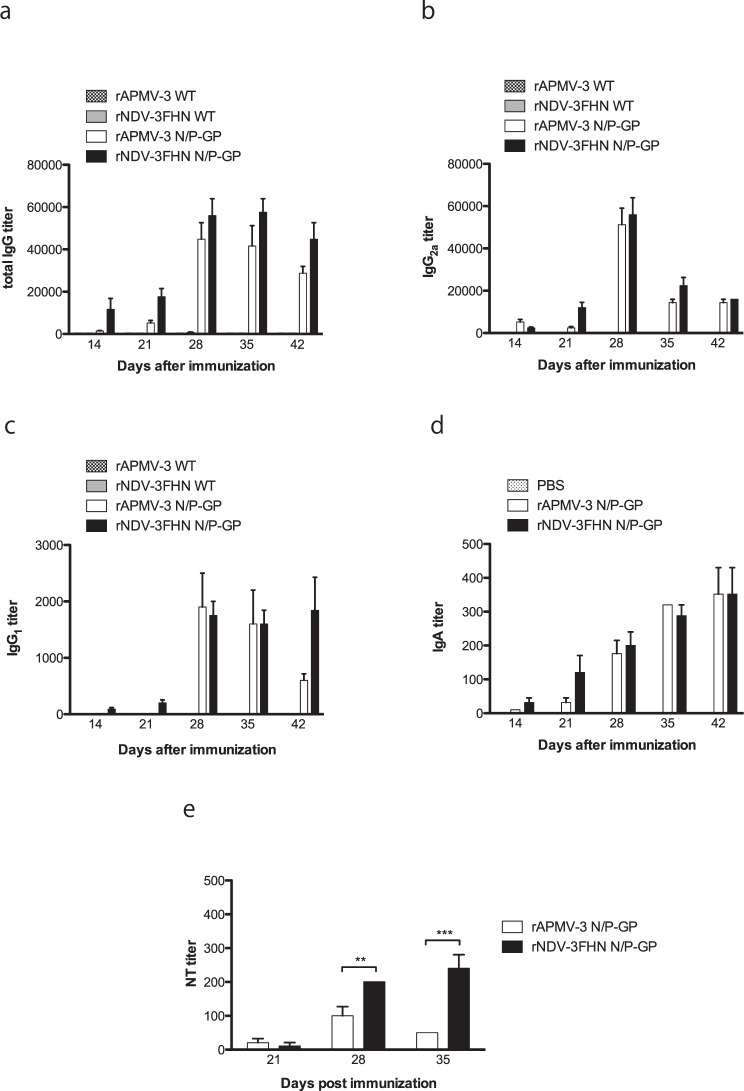
EBOV GP-specific systemic and mucosal immune responses in guinea pigs after immunization with GP-expressing recombinants and 50% plaque reduction neutralization assay. Guinea pigs were immunized with indicated GP-expressing recombinants via intranasal route twice at three weeks interval. Serum samples were collected at indicated day points after first immunization. (**a**) GP-specific total IgG titers, (**b**) IgG antibody subtypes IgG_1_, (**c**) IgG_2a_ titers and (**d**) IgA titers were measured by ELISA against purified recombinant EBOV GP. The antibody titers were defined as the reciprocal of the endpoint dilution with an optical density of ≧ 0.5. (**e**) Sera from the immunized guinea pigs at 21, 28 and 35 days after first immunization were analyzed by virus neutralization assay using rVSVΔG-ZEOBV GP. Results of three independent experiments are shown, and error bars show standard deviation. **P < 0.01; ***P < 0.001 by two-way ANOVA with Bonferroni post hoc test.

We evaluated rNDV-3FHN N/P-GP and rAPMV-3 N/P-GP as potential live vaccine candidates in guinea pig, since these viruses expressed GP efficiently. Guinea pigs are highly permissive to NDV infection and NDV infected animals do not exhibit any clinical sign^32^. First, we determined titers of the serum IgG antibodies against EBOV GP in the infected guinea pigs by an ELISA (Fig. 4). At 35 days after first immunization, the IgG antibody titers reached as high as 57,600 in animals infected with rNDV-3FHN N/P-GP, while at 28 days after immunization, the IgG antibody titers of rAPMV-3 N/P-GP sample reached as high as 44,800 (Fig. 4a). The systemic EBOV GP specific IgG response was further characterized into IgG antibody subtypes IgG_2a_ and IgG_1_, respectively. The subtype immune responses induced by rNDV-3FHN N/P-GP and rAPMV-3 N/P-GP were almost identical, respectively (Fig. 4b,c). These results indicate that the rNDV-3FHN N/P-GP can induce EBOV GP-specific IgG antibodies more efficiently (P > 0.05).

Since mucosal immune responses play an important role in defending incoming viral infection, we further investigated the induction of mucosal immune response in guinea pigs by determining IgA titer in nasal swab samples. Interestingly, not only rNDV-3FHN N/P-GP but also rAPMV-3 N/P-GP could induce significant high levels of the IgA titers, as high as 352 and they were long lasting at least 42 days after the first immunization (Fig. 4d).

The neutralizing antibody titers against EBOV GP were determined by 50% plaque reduction neutralization test using the recombinant vesicular stomatitis virus (rVSV) ΔG-ZEBOV-GP, in which the glycoprotein gene G was replaced with the GP gene of EBOV (Fig. 4e). At 21 days post immunization, very little or no level of neutralization titer was observed in both rNDV-3FHN N/P-GP and rAPMV-3 N/P-GP. However, after a second immunization, both recombinants did show an increase in antibody titers, respectively. Although rNDV-3FHN N/P-GP induced ELISA IgG titers almost similar to those of rAPMV-3 N/P-GP, its neutralizing titers were significantly higher. Furthermore, only rNDV-3FHN N/P-GP showed substantial increase of the virus neutralizing titers between 28 and 35 days after the second immunization. Taken together, these results indicate that although rNDV-3FHN as well as rAPMV-3 can elicit humoral and mucosal immune responses in guinea pigs, rNDV-3FHN is superior in ability to induce neutralizing antibodies against EBOV.

## Discussion

Since EBOV was first identified in 1976, sporadic outbreaks of EBOV have been reported for more than 30 years. However, no licensed vaccines or post-exposure treatment are currently available for EBOV infection. Although replication-competent rVSV-based EBOV vaccine candidate has shown promising results in recent trials, some side effects, such as headache, subjective fever, chill and frequent viremia have been reported^33^. Therefore, there is a need to evaluate additional safe vector vaccines against EBOV infection.

APMVs possesses a number of characteristics which benefit as a vaccine vector for human use because of absence of pre-exiting immunity in human population, robust replication and ability to induce mucosal immune responses. Among the APMVs, NDV (APMV-1) vector vaccine candidates have been shown to induce protective immune responses against several human pathogens including EBOV^15,27,34,35^. Although both lentogenic and mesogenic NDV strains can be used as vaccine vectors in humans, vectors based on mesogenic strains replicate to higher titer in NHPs and induced better immune responses than vectors based on lentogenic strains^16^. However, because of disease concern in chickens, mesogenic NDV strains are now classified as Select Agent and are prohibited for use as vaccine vectors. Therefore, in this study, we have generated an alternative NDV-based vaccine vector. This vector has the replicative machinery of a mesogenic NDV strains but the antigenicity of APMV-3. The modified NDV grew to higher titer in embryonated chicken eggs similar to APMV-3. Importantly, the modified NDV vector was apathogenic to chickens.

The safety of NDV in humans has been documented in several studies where NDV has been used as an oncolytic agent^36^. Although the safety of APMV-3 in humans is not directly known, based on the safety profile in NHP it is likely to be safe in humans^19^.

Comparison of a series of GP-expressing viruses revealed that the insertion site between N and P genes is the optimal position for GP expression, which is different from GFP-expressing APMVs reported previously^20,24,37^. Hence, our results indicate that the optimal insertion site of a foreign gene to obtain high level of protein expression is not always between the P and M genes and it can vary with each foreign gene.

It has been reported for VSV that insertion of a foreign gene between N and P genes reduced viral replication whereas insertion of a foreign gene at other gene junctions did not affect the replication level. Additionally, when a foreign gene was inserted between N and P genes, viral replication returned to parental levels within two passages and expression of the foreign gene was suppressed by mutations at the end of the upstream N gene that abolished transcriptional termination^31^. In our study, insertion of GP gene between N and P genes for both rNDV-3FHN and rAPMV-3, viral replications were slightly retarded at early time point after the infection compared with their respective parental viruses, but they replicated with efficiency and reached similar titer as their respective parental viruses. Furthermore, unlike with VSV recombinants, 20 consecutive passages did not affect the stability of GP gene inserted between N and P genes, and a high level of GP expression was still observed. Moreover, GP was efficiently incorporated into the both rNDV-3FHN and rAPMV-3 virus particles.

The immunogenicity of GP-expressing NDVs in NHPs has been assessed and compared to HPIV3-based vaccine viruses previously^15^. NDV strain BC based vaccine vector induced lower level of GP-specific serum IgG titer compared to HPIV-3 based vector but the IgA and the serum EBOV neutralizing Ab titers were equal after a second dose of vaccination^15^. However, the NDV vector used in that study was based on a mesogenic strain of NDV, which is now considered a Select Agent. This study showed that the modified NDV and APMV-3 vectors induced IgA, IgG and neutralizing antibodies in guinea pigs, but the rNDV-3FHN vector induced higher levels of antibodies than the rAPMV-3 vector. The level of antibodies induced by rNDV-3FHN was almost equal to those induced by the avirulent NDV vector strain LaSota (Supplemental Fig. 3). The reason for modified NDV vector to induce higher neutralizing titer than APMV-3 vector could be due to its relatively higher level of replication in guinea pigs. However, we do not consider the level of replication of our APMV vectors to be a concern in eliciting high levels of immunogenicity in humans because we have previously shown that NDV and APMV-3 replicate well in NHPs and elicit high immunogenicity^15,19^. In this study, EBOV neutralization was evaluated by a pseudotyped VSV BSL-2 neutralization test because a standard EBOV neutralization test will require BSL-4 containment. Although high degree of correlation between EBOV BSL-4 neutralization assays and pseudotyped VSV BSL-2 fluorescence reduction neutralization test has been reported^38^, it should be noted that pseudotyped VSV neutralization test is not a live EBOV neutralization test; therefore, its results may or may not correlate with levels of anti-EBOV neutralizing antibodies in serum samples.

In conclusion, our data suggest that the newly generated GP-expressing modified NDV and APMV-3 vectors can be an alternative vaccine for immunization against EBOV alone or in combination with NDV strain LaSota vector in a heterologous prime-boost strategy. Although we have previously demonstrated that like NDV, APMV-3 can replicate efficiently in NHPs, it is important to assess the immunogenicity and protection efficacy of these recombinant APMVs in NHPs. Taken together, the rNDV-3FHN and rAPMV-3 have great potential for use as vaccine vectors, which overcomes many weaknesses of the previously-reported paramyxoviral vaccine vectors.

## Methods

### Cells, viruses and antibodies

HEp-2, Vero and DF-1 cells were obtained from the American Type Culture Collection (Manassas, VA). The cell lines were grown in Dulbecco’s minimal essential medium (DMEM) supplemented with 10% fetal bovine serum (FBS). A modified vaccinia virus Ankara strain (MVA) expressing T7 RNA polymerase was kindly provided by Dr. Bernard Moss (NIH, Bethesda, MD). Replication-restricted rVSV that contains EBOV GP instead of the G gene (rVSVΔG-EBOV GP) was kindly provided by Dr. Heinz Feldmann (NIAID, NIH). The polyclonal antibody (pAb) against EBOV GP (IBT Biosevices, Gaithersburg, MD), the horseradish peroxidase (HRP)-conjugated anti-rabbit IgG goat antibodies (Abs) (Life Technologies and KPL, Gaithersburg, MD, respectively), and HRP-conjugated goat Abs against guinea pig IgG and IgA (KPL, and Immunology Consultants Laboratory, Newberg, OR, respectively) were used according to the protocols of the suppliers.

### Construction and recovery of recombinant viruses

The synthesized EBOV GP (strain Mayinga subtype Zaire, GenBank accession number: AY142960) cDNA (GenScript), which editing site of mRNA was modified to express the membrane-bound full-length GP, but not the sGP, flanked with the NDV^39^ or APMV-3 (GenBank accession number: EU403085) gene start and gene stop sequence motifs were inserted in the full-length cDNA genome clones of NDV and APMV-3. In NDV-3FHN cDNA, the ectodomain of NDV F gene (4,544–6,043 nt) was changed to that of APMV-3 (4,765–6,228 nt) and the ectodomain of NDV HN gene (6,550–8,145 nt) was changed to that of the APMV-3 (6,798–8,396 nt). All recombinant viruses followed the rule of six and were recovered as described previously^40^. The introduced GP genes were confirmed by DNA sequencing of the RT-PCR products using the viral genomes isolated from virus particles as templates. Viral titers of the recombinant viruses were determined as described previously^40^, and represented as the number of plaque-forming units (PFU)/ml. Multicycle growth kinetics of the recombinant viruses both in ten-day-old embryonated chicken eggs and in DF-1 cells were determined as described previously^40,41^.

### Analysis of EBOV GP expression and incorporation into virus particles

DF-1 cells cultured in 6-well plates were infected with the indicated viruses at an M.O.I. of 0.01. At 24 h p.i., cells were lysed with cell lysis buffer. Cellular expression of EBOV GP was analyzed by SDS-PAGE followed by Western blotting using anti-GP pAb. For analysis of the incorporation of EBOV GP into virus particles, the indicated viruses were inoculated into specific-pathogen-free (SPF) embryonated chicken eggs. After incubation for 72 h at 37 °C, allantoic fluids were collected, clarified by centrifugation at 3,000 rpm for 10 min., and viruses were pelleted by ultracentrifugation through a 30% sucrose cushion at 25,000 rpm for 2 h. The virus pellets were suspended in phosphate buffer saline (PBS). Virus protein profiles were analyzed by SDS-PAGE followed by CBB staining and Western blotting using anti-GP pAb.

### Pathogenicity of recombinant viruses in embryonated chicken eggs and in 1-day-old chickens

All of the animals used in this study were housed in isolator cages in our USDA approved enhanced biosafety level-2 (BSL-2+) facility following the guidelines, and approval of the Institutional Animal Care and Use Committee (IACUC), University of Maryland. All experiments were approved by IACUC and conducted following the guidelines. The pathogenicity of recombinant viruses was determined by MDT test in 9-day-old embryonated chicken eggs and by the ICPI test in 1-day-old SPF chickens^14^.

### Guinea pig immunization

Groups of five 4-week-old female Hartley guinea pigs (Charles River Laboratories, Wilmington, MA) were immunized on days 0 and 21 with 200 μl of allantoic fluid containing 10^7^ PFU/ml of the indicated virus by the intranasal route after inhalational anesthesia. After inoculation, the guinea pigs were observed daily for any clinical sign. Nasal swab samples and blood were collected on day 0 (pre-bleed) and on day 14, 21, 28, 35, and 42, and then sera were separated from the blood samples.

### Quantitative determination of GP-specific IgG, subclass IgG and IgA

EBOV GP-specific antibody titers were measured by an enzyme-linked immunosorbent assay (ELISA). 96-well Maxisorp ELISA plates (Nunc, Denmark) were coated with 50 ng/well of purified recombinant EBOV GP protein (IBT Biosevices, Gaithersburg, MD) in sodium carbonate-bicarbonate buffer at 4 °C for over-night, and blocked with 3% skim milk in PBS for 1 h. Sera and nasal swab samples were serially diluted in a series of 2-fold dilution, and added to the plate, and incubated for 2 h. The plates were washed with PBS containing 0.05% Tween-20 and incubated with HRP-conjugated anti-guinea pig IgG goat Ab or anti-guinea pig IgA sheep Ab for 1 hr. The plates were washed and developed with ABTS (2, 2′-azinobis [3-ethylbenzothiazoline-6sulfonic acid]-diammonium salt) peroxidase substrate solution. Analysis was performed at 405 nm using an ELx800 ELISA plate reader (BioTek, Winoski, VT), and the specific antibody titers were defined as the reciprocal of the endpoint dilution with an optical density of ≧0.5.

### EBOV GP neutralization assay

Serum titers of EBOV GP-specific neutralizing antibodies were determined by a 50% plaque reduction neutralization test on Vero cells using rVSVΔG-ZEBOV GP. Sera were inactivated at 56 °C for 30 min and 2-fold serial dilutions were made in serum-free DMEM, and 50 μl was incubated with 100 PFU of rVSVΔG-ZEBOV GP in total volume of 100 μl. After 1 h incubation at 37 °C, the 100 μl of serum-virus mixture was used to inoculate onto Vero cells cultured in 12-well plate, and cells were incubated for 1 h at 37 °C. Cells were then incubated with DMEM containing 0.8% methylcellulose and 2% serum. After incubation for 72 h at 37 °C, cells were fixed and stained with 1% crystal violet.

## Supplementary information

Supplemental Figures
