# Supplementary material for: Novel avian paramyxovirus-based vaccine vectors expressing the Ebola virus glycoprotein elicit mucosal and humoral immune responses in guinea pigs
Source: Sci Rep. 2019 Apr 2;9:5520. doi: 10.1038/s41598-019-42004-4 (PMC6445115; doi:10.1038/s41598-019-42004-4)
Supplement: Supplementary file 1 — Supplemental Figures [file 41598_2019_42004_MOESM1_ESM.pdf]

Novel avian paramyxovirus-based vaccine vectors expressing the Ebola virus glycoprotein elicit mucosal and humoral immune responses in guinea pigs

Asuka Yoshida, Shin-Hee Kim, Vinoth K. Manoharan, Berin P. Varghese, Anandan Paldurai and Siba K. Samal

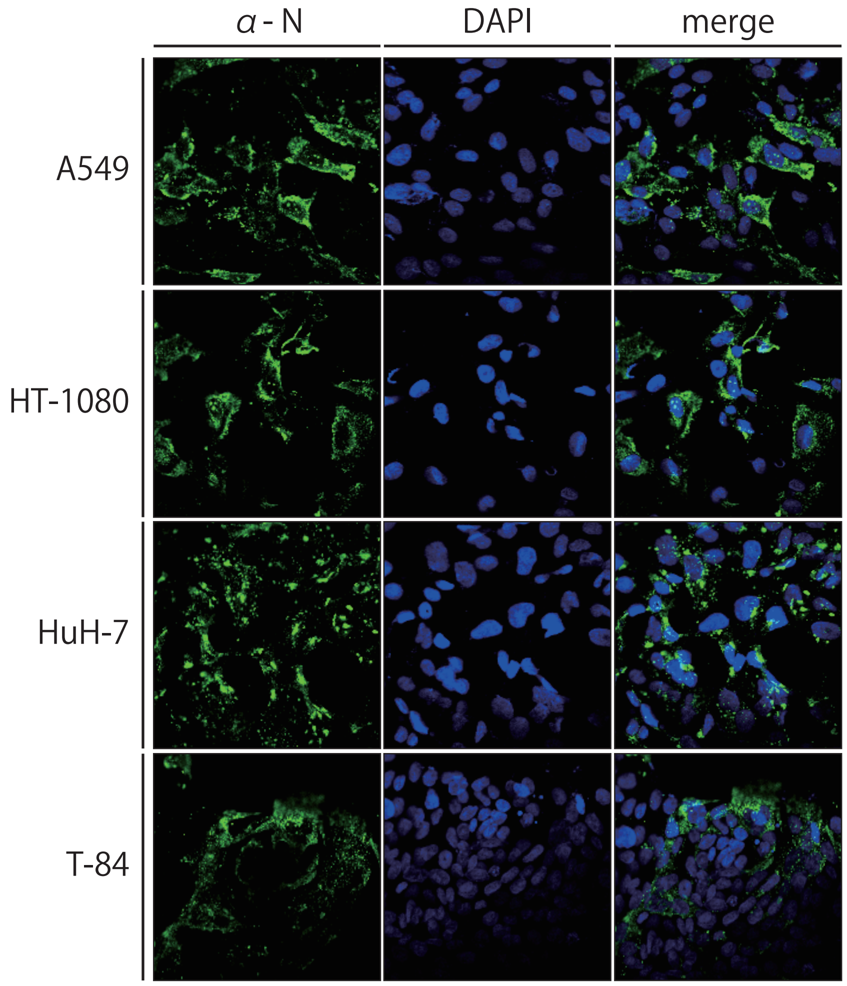

Supplemental Fig. 1: Detection of replication of APMV-3 strain Netherlands in defferent human cell lines by immunofluorescence. Different human cell lines, A549 (Human lung carcinoma), HT-1080 (Human fibrosarcoma), HuH-7 (Human hepatocellular carcinoma) and T-84 (Human colorectal carcinoma) were infected with APMV-3 strain Netherlands. After 48 h, the cells were fixed with 4% paraformaldehyde and permeabilized with Triton X-100. Rabbit anti-APMV-3 N polyclonal antibody was used as the primary antibody and goat anti-rabbit Alexa Fluor488 used as the secondary antibody conjugate. The image was captured in Zeiss LSM 510 Confocal Microscope System

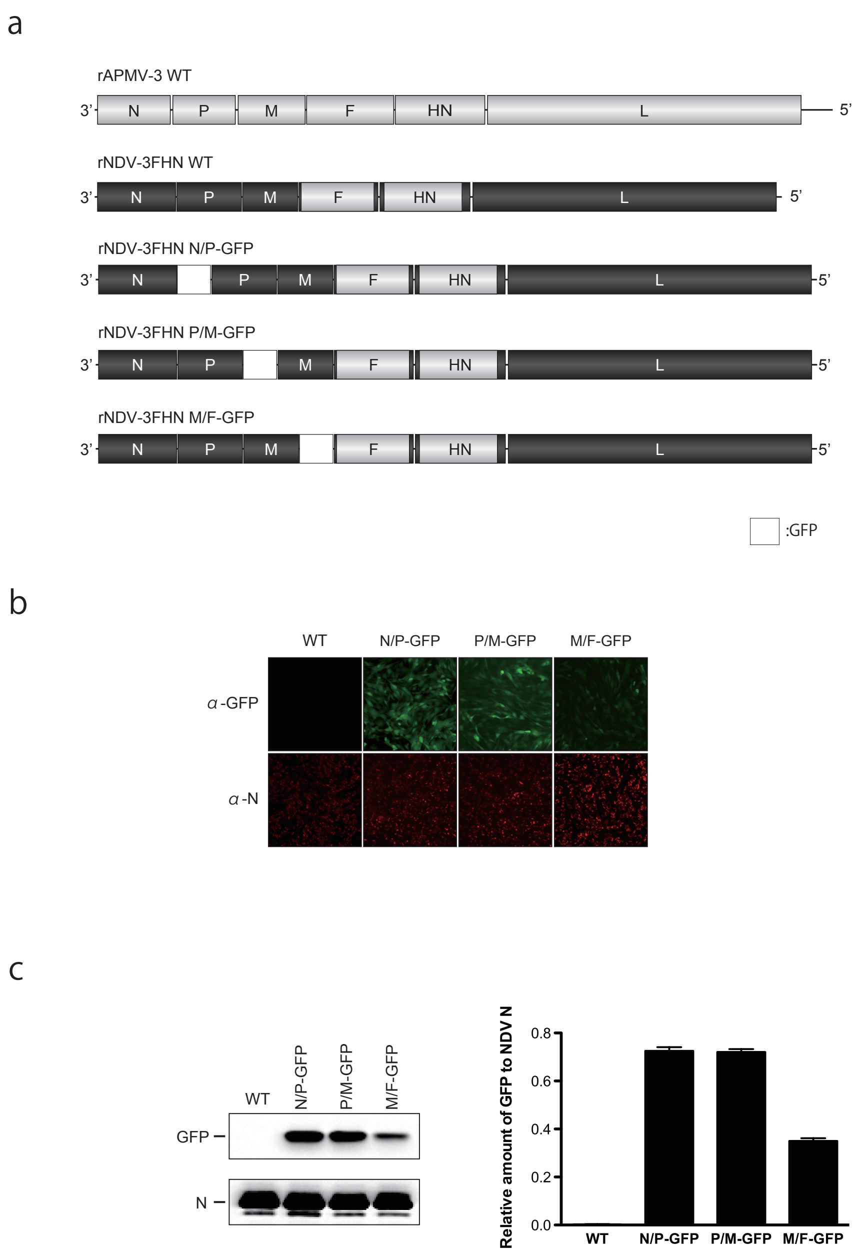

Figure S1. Fluorescent intensity and cellular expression of GFP by rNDV-3FHN recombinants . (A) Schematic representation of parental virus and rNDV-3FHN recombinants expressing GFP. GFP gene was flanked by gene start and gene end signals of NDV and inserted into indicated intergenic regions of a modified NDV antigenomic cDNA. The genes derived from NDV, APMV-3 and GFP are shown as balck, gray and white, respectively. (B) DF-1 cells were infected with modified NDV recombinant viruses at an M.O.I of 0.5. At 24 h p.i., cells were immunostained with anti-NDV N pAb as primary Abs, and Alexa Fluor 546-conjugated anti-rabbit IgG Abs as secondary Abs. GFP fluorescence was visualized under a fluorescent microscope. (C) DF-1 cells were infected with modified NDV recombinant viruses at an M.O.I of 0.5. At 24 h p.i., cells were harvested and the amount of GFP in infected cells was analyzed by SDS-PAGE and followed by Western blotting with anti-GFP pAb. Amount of N proteins and GFP proteins were quantitated and the ratio of GFP to N is shown as bar graph.

a

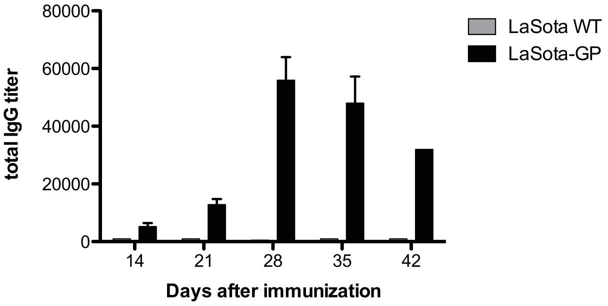

b

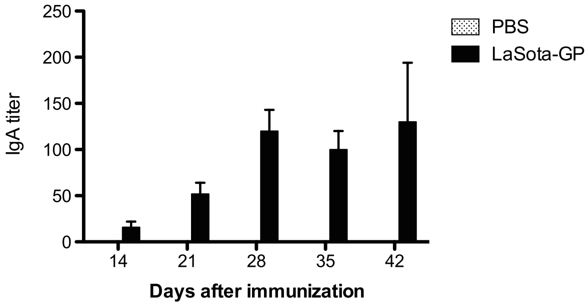

Figure S2. EBOV GP-specific systemic IgG and mucosal immune response in guinea pigs after immunization with EBOV GP expressing NDV strain LaSota. Guinea pigs were immunized with GP expressing NDV strain LaSota via intranasal route twice at three weeks interval. Searum and nasal swab samples were collected at indicated day points after first immunization. (A) EBOV GP-specific total IgG titers and (B) IgA titers were measured by ELISA against purified recombinant EBOV GP. The antibody titers were defined as the reciprocal of the endpoint dilution with an optical density of ≧0.5
